# Impact of the COVID-19 pandemic on urgent dental care delivery in a Swiss university center for dental medicine

**DOI:** 10.1007/s00784-021-03872-1

**Published:** 2021-03-12

**Authors:** Florin Eggmann, Asin Ahmad Haschemi, Dimitrios Doukoudis, Andreas Filippi, Carlalberta Verna, Clemens Walter, Roland Weiger, Nicola U. Zitzmann, Michael M. Bornstein

**Affiliations:** 1 Department of Periodontology, Endodontology and Cariology, University Center for Dental Medicine UZB, University of Basel, Mattenstrasse 40, CH-4058 Basel, Switzerland; 2 Department of General Pediatric and Adolescent Dentistry, University Center for Dental Medicine UZB, University of Basel, Basel, Switzerland; 3 Department of General Dentistry, University Center for Dental Medicine UZB, University of Basel UZB, Basel, Switzerland; 4 Department of Oral Surgery, University Center for Dental Medicine Basel UZB, University of Basel, Basel, Switzerland; 5 Department of Pediatric Oral Health and Orthodontics, University Center for Dental Medicine Basel UZB, University of Basel, Basel, Switzerland; 6 Department of Reconstructive Dentistry, University Center for Dental Medicine Basel UZB, University of Basel, Basel, Switzerland; 7 Department of Oral Health & Medicine, University Center for Dental Medicine Basel UZB, University of Basel, Basel, Switzerland

**Keywords:** SARS-CoV-2, Coronavirus infection, Dentistry, Dental public health, Access to care

## Abstract

**Objectives:**

This study aimed to assess whether the emergency service of a major Swiss dental institution faced different demands (patient volume, treatment needs, dental care characteristics) during a lockdown, issued to mitigate the COVID-19 pandemic, compared with the weeks before and after.

**Materials and methods:**

Data of patients receiving urgent care at a university center for dental medicine (Basel, Switzerland) during the 6-week lockdown, pre-lockdown, and post-lockdown periods were retrospectively evaluated. Statistical analysis involved tests for equal proportions and logistic regression models. The level of significance was set at *α*=0.05.

**Results:**

The study comprised 3109 dental emergency visits in the period from February 2 to June 5, 2020. Daily caseloads increased during lockdown. Abscesses, orthodontic emergencies, and surgical follow-ups were more common during lockdown, whereas the number of dento-alveolar injuries declined (≤0.048). Urgent dental care provision involved intraoral radiographs more frequently in the pre-lockdown period compared with the following weeks (*p*<0.001). Among all treatments, aerosol-generating procedures dropped from 56.1% (pre-lockdown) to 21.3% during lockdown (*p*<0.001), while teledentistry follow-ups became more frequent (*p*<0.001). Patients with comorbidities sought urgent dental care less frequently during the post-lockdown period (*p*=0.004).

**Conclusions:**

The lockdown significantly impacted the dental emergency service in terms of patients’ diagnoses, treatment needs, and the characteristics of the urgent care that was delivered.

**Clinical relevance:**

Access to essential dental care must be monitored and safeguarded throughout the COVID-19 pandemic and beyond as deferred care entails risks for serious sequelae and persons with comorbidities may change their dental care-seeking behavior.

## Introduction

The delivery of dental care regularly involves close contact to patients, exposure to blood and saliva, and procedures that produce droplets and aerosols. Consequently, it may entail a heightened risk for cross-infection with severe acute respiratory syndrome coronavirus 2 (SARS-CoV-2), the causative agent of the coronavirus disease 2019 (COVID-19) [1, 2]. Based on current evidence, the risk of dental healthcare personnel (DHCP) contracting SARS-CoV-2 from asymptomatic patients is deemed low provided that DHCP wear advanced personal protective equipment (PPE) including N95 respirators (or equivalent masks) [3, 4].

The appropriate management of dental emergencies is crucial to alleviate the burden faced by hospital emergency departments [5]. The timely and major reorganization of dental care services is, however, a challenge, and published data on utilization of dental emergency services during this pandemic are still limited [2, 6–8]. In mainland China, the use of teledentistry services increased considerably during lockdown [8]. A retrospective study from the School and Hospital of Stomatology at Wuhan University showed that endodontic pain was the most common reason for patients’ visits during the pandemic [6]. Dental emergency services in Beijing, China, saw a 38% decrease in first-visit patients seeking urgent dental care and the number of dento-alveolar injuries showed a significant decline [7].

To curb the spread of SARS-CoV-2, the Swiss Federal Council gradually imposed public health measures which included restrictions for dental care services. Dental practitioners had to defer or suspend treatments that were not considered urgent in the 6-week period from March 17, 2020, to April 27, 2020. The University Center for Dental Medicine Basel (UZB), a major tertiary center for dental medicine, was responsible for delivering urgent dental care to COVID-19 patients in the northwest of Switzerland (5/26 cantons of Switzerland). Thus far, its emergency service has remained open for patients with urgent dental treatment needs throughout the COVID-19 pandemic.

Until an effective treatment or a safe and effective widespread immunization program or both are available, populations remain highly vulnerable to SARS-CoV-2 infections and the resulting disease. When one wave of COVID-19 has abated, there is a substantial risk for viral reintroduction or resurgence and possible subsequent waves ought to be anticipated [9]. Therefore, further research into the impact of the COVID-19 pandemic on dental emergency services is urgently needed to enhance the critical preparedness required to safeguard the efficient and safe provision of essential dental care throughout the current global health crisis.

The aim of this study was therefore to retrospectively evaluate the dental emergency services that were provided at the UZB during the 6-week lockdown period of wide-ranging public health measures issued by the Federal Council (16 March [starting a midnight] until 26 April 2020). Data on urgent dental care delivered in the 6-week period before and after 17 March and 26 April 2020, respectively, were used to determine if the dental emergency service faced different demands at distinct stages of the pandemic. The demand faced by dental emergency service was defined by the volume and composition of patients seeking urgent care, their treatment needs, and the treatment modalities used in the provision of urgent dental care. Given the urgency of the COVID-19 health crisis and the singularity of the federal lockdown measures, a retrospective study design was chosen. The null hypothesis was that the three time periods assessed would exhibit no difference regarding the patient population, patients’ treatment needs, and the characteristics of urgent dental care provision.

## Materials and methods

### Ethical approval

The study was conducted in accordance with the regulatory requirements of the Swiss Human Research Act and Human Research Ordinance and it complied with Good Clinical Practice principles. The local Ethics Committee (EKNZ) approved this retrospective study (EKNZ 2020-01512).

### Informed consent

On their first visit, patients treated at the UZB received information and General Consent (GC) documents from the admission staff or other UZB employees. Patients gave their informed GC by signing the UZB GC form.

### Setting

Located in Basel, Switzerland’s third most populous city with about 179,000 inhabitants, the UZB is a major tertiary center for dental medicine. In 2019 and 2020, the UZB recorded a total of 85,800 and 72,500 dental patient consultations, respectively.

### Restrictions in dental care and infection prevention and control practices

During the COVID-19 pandemic, along standard hygiene practices, the UZB has implemented additional infection prevention and control practices, which are outlined in Table 1. Elective procedures and non-urgent visits were suspended or postponed in the period from 17 March until 26 April 2020. Only staff members involved in the provision of urgent dental care, including administrative work, facility management, sterile processing, and cleaning, were present at the UZB. The rest of the workforce worked from home or was furloughed. Elective procedures and non-urgent visits were gradually resumed after 27 April 2020. Additional infection prevention and control practices have remained in place ever since.

**Table 1:** Overview of additional infection prevention and control practices implemented at the UZB during the COVID-19 pandemic

Domain	Infection prevention and control measures
Workforce	Only core of essential, healthy staff present at the UZB
	Mandatory self-screening for symptoms consistent with COVID-19, exposure to others with COVID-19, and fever (>37.5°C) before going to work
	Mandatory social distancing in non-patient care areas
	Strict hand hygiene
Screening and triage	*Patients contacting by phone*
	Screening for COVID-19, symptoms consistent with COVID-19, exposure to others with COVID-19, and fever (>37.5°C)
	Assessment of the urgency of the dental treatment need
	Use of teledentistry options whenever viable*
	*Patients presenting at the UZB*
	Traffic light system outside the patient entrance to ensure social distancing
	Compulsory ABHR and type II surgical mask, supplied at no charge
	Triage check-in and reception equipped with physical barriers
	Screening for fever (>37.5°C) with a contactless forehead thermometer
	Screening questionnaire (screening for COVID-19, symptoms consistent with COVID-19, exposure to others with COVID-19, and fever)
	Targeted medical history by DHCP and triage (urgent dental care needed, dental care can be deferred, immediate referral to a hospital warranted)
	Visitors limited to caregivers/parents essential for a patient’s care and/or well-being
PPE	Donning and doffing of PPE according to CDC recommendations
	*General PPE*
	Mandatory mask-wearing for patients and staff
	KN95 respirators for DHCP, type IIR surgical masks for other staff members, type II surgical masks for patients
	*PPE for dental care delivery*
	Scrubs, type IIR surgical mask worn over a KN95 respirator, disposable surgical head cap, protective glasses, face shield, medical gloves
	*PPE for dental care delivery for patients confirmed/suspected COVID-19*
	Operating room restricted shoes, disposable surgical trousers, gowns and caps, type IIR surgical mask worn over a KN99 respirator, protective glasses, face shield, surgical gloves
Waiting areas and restrooms	Mandatory social distancing
	Separate waiting areas and restrooms for patients with confirmed/suspected COVID-19
	No-touch dispensers of ABHR and receptacles for disposal of tissues and suchlike at the entrance, the reception, the waiting areas and the patient rooms
	Chairs in the waiting areas positioned >2 m apart
	No newspapers, magazines, toys in the waiting areas
	Placards issued by the Federal Office of Public Health with instructions on social distancing, hand hygiene, and cough etiquette
	Regular wipe disinfection
Patient rooms	Individual patient rooms
	Access restricted to DHCP essential for patient care
	Regular impact ventilation where possible
	Dedicated containers for PPE and any waste
	Environmental cleaning and disinfection procedures after each patient
	High-level disinfectants
	For patients with confirmed/suspected COVID-19, designated patient room equipped with a high-efficiency particulate air filtration system
	Fallow period ≥30 min after environmental cleaning and disinfection before re-entry into that patient room was allowed
Urgent dental care	Preprocedural mouth rinse (hydrogen peroxide 1%, 30 s)
	Instructions for DHCP:
	- Exclusively use hand instruments whenever possible
	- Avoid aerosol generating procedures (high-speed handpieces, air/water syringes) whenever possible
	- Avoid procedures that may trigger coughing or increase the salivary flow whenever possible
	- Use rubber dam whenever feasible, place it so that it covers the patient’s nose and disinfect the operative field
	- Take intraoral radiographs only when absolutely necessary and consider extraoral imaging (panoramic radiographs, CBCT) as preferential alternative
	- One-visit treatment whenever possible
	Prohibition on the use of turbines, ultrasonic instruments, and air-polishing devices
	Mandatory use of four-handed dentistry and high volume suction during aerosol generating procedures

*ABHR*, alcohol-based hand rub; *CBCT*, cone-beam computed tomography; *CDC*, Centers for Disease Control and Prevention; *COVID-19*, coronavirus disease 2019; *DHCP*, dental healthcare personnel; *PPE*, personal protective equipment; *UZB*, University Center for Dental Medicine Basel

*Teledentistry at the UZB involved phone or email consultations, mainly focusing on the assessment of the treatment need including its urgency and patient counseling in cases of a non-urgent treatment need (for example, management of dentin hypersensitivity with over-the-counter products)

### Data retrieval

The three periods, pre-lockdown, lockdown and post-lockdown, each comprised 27 working days (Monday to Friday) during which urgent dental care was delivered. The timeframes of the three periods used for data retrieval were as follows: pre-lockdown, 07 February 2020 until 16 March 2020; lockdown, 17 March 2020 until 24 April 2020; and post-lockdown, 27 April 2020 until 06 June 2020. For the retrospective assessment, personal and medical data, including medical imaging data, of patients receiving urgent dental care at the UZB in the timeframe from 07 February 2020 until 05 June 2020 were used.

Inclusion criteria were the following:

- Utilization of urgent dental care (including related follow-up consultations after urgent dental care) at the UZB in the period from 07 February 2020 until 05 June 2020.

Exclusion criteria were the following:

- Data from patients who received scheduled, routine, elective dental care were not used (i.e., before and after the six-week lockdown period).

With data encoding implemented from the outset, data were retrieved in a standardized manner from the individual case files of all patients who were eligible according to the inclusion/exclusion criteria. No additional data were acquired for research purposes. The dataset extracted comprised patient characteristics with epidemiological background data (age, gender, registration status, length of the journey to the UZB, underlying medical conditions increasing the risk for severe illness from SARS-CoV-2, SARS-CoV-2 status), characteristics of the main urgent dental health problems, and characteristics of the urgent care. The dataset generated and analyzed during the current study is available from the corresponding author on request. Table 2 provides an overview of the data items collected from eligible patients’ case files.

**Table 2:** Set of data extracted from eligible patients’ case files (categories where applicable given in brackets)

Patient characteristics	Characteristics of a patient’s main dental health problem	Characteristics of the dental care delivered to a patient
Age	Acute pain	Radiologic assessment (none/yes)
Gender (female/male/other)	Abscess lesion	Intraoral radiograph (no/yes)
Registration status (first time patient/registered patient)	Dento-alveolar injury	Panoramic radiograph (no/yes)
Length of journey to the UZB (km)*	Facial pain/TMJ disorder	CBCT (no/yes)
Underlying medical condition (none/one or more)	Hemorrhagic incident	Pharmacologic management (no/yes)
Chronic lung disease and/or moderate/severe asthma (no/yes)	Lesion of the oral mucosa	Medication (none/analgesics/analgesics and antibiotics/antibiotics/other)
Serious heart condition (no/yes)	Orthodontic emergency	Aerosol generating procedure (no/yes)
Immunocompromised patient (no/yes)	Pre-surgery/pre-radiotherapy/pre-ART care	Rubber dam (no/yes)
Diabetes (no/yes)	Prosthodontic emergency	Restorative treatment (no/yes)
Chronic kidney disease undergoing dialysis(no/yes)	Restoration fracture	Endodontic treatment (no/direct pulp capping/partial pulpotomy/pulpotomy/root canal treatment)
Liver disease (no/yes)	Surgical follow-up (suture removal, drain removal/renewal etc.)^†^	Periodontal treatment (no/debridement/NUG or NUP treatment)
Cancer (no/yes)		Extraction (no/dental implant removal/simple tooth extraction/surgical tooth extraction)
SARS-CoV-2 status (known SARS-CoV-2 infection/recovered from COVID-19/suspected SARS-CoV-2 infection/unknown SARS-CoV-2 infection status		Abscess treatment (no/yes)
		Dentitio difficilis treatment (no/yes)
		Splinting (no/yes)
		Follow-up treatment (no/yes)
		Prosthodontic emergency treatment (no/yes)
		Oral medicine treatment (no/yes)
		Orthodontic emergency treatment (no/yes)
		Examination/counseling (no other treatment) (no/yes)
		Follow-up over phone (no/yes)
		Referral to hospital (no/yes)

*CBCT*, cone-beam computed tomography; *COVID-19*, coronavirus disease 2019; *NUG*, necrotizing ulcerative gingivitis; *NUP*, necrotizing (ulcerative) periodontitis; *SARS-CoV-2*, severe acute respiratory syndrome coronavirus 2; *pre-ART care*, dental care prior to antiresorptive therapy; *TMJ*, temporomandibular joint; *UZB*, University Center for Dental Medicine Basel

*Measured, in Google maps, as the car journey shortest in length from a patient’s home address to the UZB

^†^Exclusively surgical follow-ups related to a preceding urgent dental treatment were considered

Among comorbidities, exclusively underlying medical conditions that increase the risk for severe illness from SARS-CoV-2 were taken into account

### Statistical analysis

The statistical analysis included descriptive statistics of the patient population receiving urgent dental care and of the treatments that were provided during the three time periods (prior to, during, and after the lockdown period) with the corresponding overall significance tests. Logistic regression models adjusted for age and gender were performed in order to predict the binomial data structure (e.g., yes versus no) of the clinical parameters. Pairwise comparisons were performed by the subsequent analysis by Tukey contrasts. The level of significance was set at *α* = 0.05. All analyses were carried out with the statistical program R version 3.5.1 (R Foundation for Statistical Computing, Vienna, Austria).

## Results

The study included 3109 dental emergency visits of eligible patients who sought urgent dental care in the entire study period from 07 February 2020 until 05 June 2020. The demand for urgent dental care saw an increase during lockdown: The daily patient volume in the emergency service was, on average, 32.9 in the pre-lockdown period, 41.5 in the lockdown period, and 40.8 in the post-lockdown period. The gender balance of patients seeking urgent dental care was slightly skewed towards males. The patients’ mean age, 43.7 years across the three periods, showed no significant differences (*p*=0.285). The average length of the journey from the patients’ residence to the UZB increased during lockdown compared with the periods before and after (*p*<0.001). The percentage of patients with underlying medical conditions decreased over the observation period: Fewer patients with one or more comorbidities sought urgent dental care during the lockdown (*p*=0.695) and the post-lockdown period (*p*=0.004). One patient with confirmed and five with suspected COVID-19 were treated during the lockdown period. Table 3 shows the patients’ characteristics in detail.

**Table 3:** Patient characteristics in the three periods, pre-lockdown, lockdown, and post-lockdown (unless otherwise stated, data are presented as absolute numbers with the corresponding percentage given in brackets)

Patient characteristics	Period				
	Pre-lockdown	Lockdown	Post-lockdown	Statistical test(s)	*p* value
Age				Kruskal-Wallis test	0.285
Median (IQR)	46.9 (28.4, 60.4)	43.9 (25.6, 60.1)	45 (25.8, 61.5)		
Mean (SD)	44.5 (21.6)	43 (22)	43.7 (22.7)		
Gender				Chi-squared test	1
Female	430 (48.5)	543 (48.4)	533 (48.4)		
Male	457 (51.5)	578 (51.6)	568 (51.6)		
Length of journey (km)				Kruskal-Wallis test	<0.001
Median (IQR)	2.9 (1.6, 4.4)^A^	3.3 (2.2, 5.6)^B^	3 (1.4, 4.6)^A^		
Mean (SD)	6 (13.4)^A^	9.1 (24.2)^B^	5.7 (13.1)^A^		
Underlying medical condition (one or more)	226 (25.5)^A^	260 (23.2)^A^	209 (19)^B^	Chi-squared test and Tukey contrasts of the logistic regression models	0.002
Chronic lung disease and/or moderate/severe asthma	47 (5.3)	50 (4.5)	35 (3.2)		0.06
Serious heart condition	108 (12.2)^A^	108 (9.6)^A^	86 (7.8)^B^		0.005
Immunocompromised patient	18 (2)	18 (1.6)	10 (0.9)		0.109
Diabetes	78 (8.8)^A^	57 (5.1)^B^	80 (7.3)^A,B^		0.004
Chronic kidney disease undergoing dialysis	1 (0.1)	2 (0.2)	4 (0.4)		0.462
Liver disease	14 (1.6)	23 (2.1)	11 (1)		0.131
Cancer	35 (3.9)^A^	62 (5.5)^A^	20 (1.8)^B^		<0.001
SARS-CoV-2 status					0.095
Known SARS-CoV-2 infection	0 (0)	1 (0.1)	0 (0)		
Recovered from COVID-19	0 (0)	1 (0.1)	5 (0.5)		
Suspected SARS-CoV-2 infection	2 (0.2)	5 (0.4)	1 (0.1)		
Unknown SARS-CoV-2 infection	885 (99.8)	1114 (99.4)	1095 (99.5)		

*COVID-19*, coronavirus disease 2019; *IQR*, interquartile range; *SARS-CoV-2*, severe acute respiratory syndrome coronavirus 2; *SD*, standard deviation

Within the same row, different superscript letters indicate significant differences in the Tukey contrasts

The percentage of patients with acute pain, the main reason for seeking urgent dental care throughout the observation period, was significantly lower during lockdown compared with the pre- and post-lockdown periods (*p*≤0.007). Dental abscesses, orthodontic emergencies, and surgical follow-ups were more common during lockdown. During lockdown, the number of dento-alveolar injuries decreased (≤0.048). The caseload of dento-alveolar injuries was highest in the weeks after lockdown. Restoration fracture and prosthodontic emergencies were a less common reason to seek urgent dental care during lockdown (≤0.013). A significant drop in patients seeking preparatory dental measures (pre-surgery/pre-radiotherapy/pre-ART dental care) was observed during lockdown und post-lockdown (*p*≤0.021). The characteristics of patients’ main dental health problems are provided in Table 4.

**Table 4:** Characteristics of patients’ main dental health problems (given in absolute numbers and in brackets as percentage)

Main dental health problem	Period			Statistical tests	*p* value
	Pre-lockdown	Lockdown	Post-lockdown		
Acute pain	462 (52.1)^A^	508 (45.3)^B^	563 (51.1)^A^	Pairwise tests for equal proportions between the period groups	≤0.007
Abscess lesion	30 (3.4)^A^	68 (6.1)^B^	40 (3.6)^A^		≤0.01
Dento-alveolar injury	39 (4.4)^A^	30 (2.7)^B^	57 (5.2)^A^		≤0.048
Facial pain/TMJ disorder	6 (0.7)	14 (1.2)	9 (0.8)		≥0.291
Hemorrhagic incident	2 (0.2)	3 (0.3)	2 (0.2)		n. d.
Lesion of the oral mucosa	23 (2.6)	22 (2)	30 (2.7)		≥0.295
Orthodontic emergency	2 (0.2)^A^	37 (3.3)^B^	11 (1)^A^		<0.001
Pre-surgery/pre-radiotherapy/pre-ART care	29 (3.3)^A^	18 (1.6)^B^	13 (1.2)^B^		≤0.021
Prosthodontic emergency	122 (13.8)^A^	113 (10.1)^B^	124 (11.3)^A,B^		0.013
Restoration fracture	92 (10.4)^A^	11 (1)^B^	92 (8.4)^A^		<0.001
Surgical follow-up	80 (9)^A^	297 (26.5)^C^	160 (14.5)^B^		<0.001

*n. d.*, not determined; *TMJ*, temporomandibular joint; *pre-ART care*, dental care prior to antiresorptive therapy

Within the same row, different superscript letters indicate significant differences between the period groups

Urgent dental care provision involved intraoral radiographs more frequently in the pre-lockdown period compared with the following weeks (*p*<0.001). No shift towards a more frequent use of extraoral imaging was observed over time. Pharmacological management was more frequent during lockdown (7.4%) in comparison with the period after (4.6%) (*p*=0.018). During lockdown, 21.3% of all treatments involved aerosol-generating procedures, a drop of 34.8% compared with the pre-lockdown period (*p*<0.001). Rubber dam was more frequently used during lockdown compared with the post-lockdown period (*p*=0.004). Restorative and periodontal treatments (except for treatment for necrotizing periodontal diseases) were less common in the lockdown period. The lockdown period saw an increase in abscess treatments, simple tooth extractions, surgical follow-ups, and treatments for necrotizing periodontal diseases. Telephone follow-ups, rare across the three periods, showed a slight increase during lockdown (<0.001). During the lockdown and post-lockdown period, four patients with severe fascial space infections requiring hospitalization were recorded, while no such infection was observed before lockdown (*p*≥0.20). Detailed characteristics of the dental care delivered are reported in Table 5.

**Table 5:** Characteristics of the dental care procedures delivered to patients (data are presented as absolute numbers with the corresponding percentage given in brackets)

Dental care	Period			Statistical tests	*p* value
	Pre-lockdown	Lockdown	Post-lockdown		
Radiologic assessment	530 (59.8)^A^	460 (41)^C^	552 (50.1)^B^	Tukey contrasts of logistic regression models	**<0.001**
Intraoral radiograph	432 (48.7)^A^	391 (34.9)^C^	480 (43.6)^B^		**<0.001**
Panoramic radiograph	156 (17.6)^A^	94 (8.4)^B^	111 (10.1)^B^		**<0.001**
CBCT	9 (1)	7 (0.6)	3 (0.3)		0.108
Pharmacologic management	49 (5.5)^A,B^	83 (7.4)^A^	51 (4.6)^B^		**0.018**
*Medication*					
Analgesics	19 (2.1)	37 (3.3)	20 (1.8)		n. d.
Analgesics and antibiotics	7 (0.8)	20 (1.8)	15 (1.4)		n. d.
Antibiotics	23 (2.6)	25 (2.2)	14 (1.3)		n. d.
Other	0 (0)	1 (0.1)	2 (0.2)		n. d.
Aerosol generating procedure	498 (56.1)^A^	239 (21.3)^B^	583 (53)^A^		**<0.001**
Rubber dam	58 (6.5)^A,B^	89 (7.9)^B^	54 (4.9)^A^		**0.005**
Restorative treatment	229 (25.8)^A^	222 (19.8)^B^	218 (19.8)^B^		**<0.001**
Endodontic treatment					0.611
Direct pulp capping	0 (0)	2 (0.2)	1 (0.1)		n. d.
Partial pulpotomy	1 (0.1)	2 (0.2)	1 (0.1)		n. d.
Pulpotomy	9 (1)	12 (1.1)	6 (0.5)		n. d.
Root canal treatment	45 (5.1)	64 (5.7)	48 (4.4)		n. d.
Periodontal treatment					
Debridement	59 (6.7)^A^	25 (2.2)^B^	74 (6.7)^A^		**<0.001**
NUG or NUP treatment	0 (0)	7 (0.6)	1 (0.1)		n. d.
Extraction					
Dental implant removal	0 (0)	0 (0)	2 (0.2)		n. d.
Simple tooth extraction	136 (15.3)^A^	211 (18.8)^A,B^	224 (20.3)^B^		**0.048**
Surgical tooth extraction	14 (1.5)	21 (1.9)	17 (1.5)		n. d.
Abscess treatment	24 (2.5)^A^	51 (4.5)^B^	25 (2.2)^A^		**0.002**
Dentitio difficilis treatment	22 (2.3)	33 (2.9)	28 (2.4)		0.589
Splinting	9 (0.9)	7 (0.6)	10 (0.9)		0.702
Surgical follow-up treatment	66 (6.9)^A^	233 (20.8)^B^	110 (9.5)^A^		**<0.001**
Prosthodontic emergency treatment	95 (9.9)	88 (7.9)	102 (8.8)		0.269
Oral medicine treatment	100 (10.4)^A^	51 (4.5)^B^	31 (2.7)^C^		**<0.001**
Orthodontic emergency treatment	2 (0.2)^A^	37 (3.3)^B^	11 (0.9)^A^		**<0.001**
Examination/counseling	225 (23.4)^A^	60 (5.4)^B^	275 (23.7)^A^		**<0.001**
Follow-up over phone*	2 (0.2)^A^	26 (2.3)^B^	3 (0.3)^A^		**<0.001**
Referral to hospital	0 (0)	3 (0.3)	1 (0.1)		0.20

*CBCT*, cone-beam computed tomography; *n. d.*, not determined; *NUG*, necrotizing ulcerative gingivitis; *NUP*, necrotizing (ulcerative) periodontitis; *TMJ*, temporomandibular joint

Within the same row, different superscript letters indicate significant differences in the Tukey contrasts

*Inquiry regarding postoperative complaints, analgesia, tolerance of prescribed drugs, and so forth. Considered a teledentistry modality at the UZB

## Discussion

This retrospective study aimed to assess the impact of the COVID-19 pandemic on urgent dental care provision in a major dental institution in Switzerland. Its results suggest that the dental emergency service faced different demands at distinct phases of the pandemic. Significant changes were observed in the composition of patients seeking urgent dental care, their treatment needs, and the delivery of urgent dental care. The provision of urgent care saw a shift towards more frequent surgical treatments and a significant decrease in aerosol-producing procedures during the lockdown period. Based on the strength of these significant differences found, the null hypothesis had to be rejected.

There were some notable changes in the composition of patients seeking urgent dental care during lockdown and in the following weeks. Though no significant differences in the average age and the gender balance were observed, data indicate that the catchment area of the UZB dental emergency service expanded during the lockdown. This was most likely due to some dental healthcare providers in the region suspending their services altogether. This underlines the importance of regional dental emergency centers for the provision of urgent care in times of crisis [10]. For instance, the substantial increase in orthodontic emergencies and surgical follow-ups indicates that, during lockdown, the availability of urgent care was limited for such patients. Moreover, fewer patients with underlying medical conditions that increase the risk for severe illness from SARS-CoV-2 sought urgent dental care during the lockdown and beyond. This suggests that the COVID-19 pandemic had a measurable impact on the dental care seeking behavior of people with underlying medical conditions. It may be assumed that some persons with comorbidities refrained from seeking dental care owing to perceived risks for SARS-CoV-2 infection in dental settings or on the way there. Thus, it is important that dental healthcare providers facilitate safe access to essential dental care to vulnerable groups.

In line with a previous study, a significant drop in dental injuries was observed during lockdown [7]. This was likely due to restrictions in athletic leisure activities and changes in commuting in that period. The uptick in dental injuries in the post-lockdown period may be explained by a probable compensatory increase in sporting activities as well as seasonally related effects on sporting and commuting behavior. Meanwhile, a significant increase in patients with abscess lesions was observed during lockdown. The increased rate of infections may reflect sequelae that occurred due to suspended and deferred dental care or a pandemic-related omission in care seeking. A recent study, based on representative US survey data, reported that 46.7% of US adults delayed going to the dentist or receiving dental care owing to the COVID-19 pandemic [11]. The present retrospective study, likewise, suggests that the pandemic led a segment of the population to delay dental care in spite of an urgent treatment need. Therefore, as long as restrictions are imposed on dental healthcare providers, it is important to identify patients to whom suspended or deferred care presents a likely health risk and to arrange timely follow-ups.

Overall, emergency periodontal treatments had to be carried out less frequently during lockdown. However, it is noteworthy that treatments for necrotizing periodontal diseases increased in the lockdown period. The appropriate management of patients with periodontitis, who frequently display only few symptoms [12], is important, in particular with respect to the COVID-19 pandemic because evidence from a case-control study suggests that the risk of COVID-19 complications is significantly higher among patients with moderate-to-severe periodontitis compared to those with milder or no periodontitis [13].

Contaminated aerosols and splatter are an important potential mode of transmission for many pathogens including SARS-CoV-2 [14, 15]. The present study showed that aerosol-generating procedures could be reduced significantly with a concomitant increase in the use of rubber dam isolation during lockdown. For aerosol-generating procedures, which mainly involved the use of water-cooled contra-angle handpieces, it was mandatory to use four-handed dentistry and assistant-held high volume suction, proven measures to reduce contamination [14, 16]. Yet, it was notable that aerosol-generating procedures and rubber dam use reached pre-lockdown levels when the most severe government-issued restrictions were lifted. This may point towards a certain degree of negligence, on the part of DHCP, in the adherence to special infection prevention and control measures when the infectious health threat was perceived to have waned.

DHCP ought to avoid procedures that may trigger patients to cough whenever possible in the COVID-19 pandemic [10]. The delivery of urgent dental care involved a radiologic assessment more frequently in the pre-lockdown period. However, there was no shift towards more frequent extraoral imaging during lockdown and the weeks that followed even though DHCP were instructed to consider panoramic radiographs and even CBCT as alternatives [10, 17]. The reasons for this finding may be twofold. First, clear guidelines during the COVID-19 pandemic notwithstanding, DHCP were likely hesitant to use extraoral imaging in cases where the diagnostic value of intraoral imaging was sufficient in order to adhere to the radioprotection principle of keeping patients’ exposure to ionizing radiation as low as reasonably achievable [18]. Second, some patient rooms were equipped to take intraoral radiographs, whereas extraoral imaging could be performed exclusively at the dental imaging department. As a way to avoid interrupting the examination or treatment and to keep patients’ movement at the UZB to a minimum, DHCP may have deemed chair-side intraoral imaging more convenient and advantageous. With strict infection prevention and control measures in place, it is difficult to judge whether intraoral radiographs taken in an individual patient room entail more risks for cross-infection compared with extraoral imaging performed elsewhere.

Strict adherence to the hygiene chain and special infection prevention and control measures are needed to reduce the risk of cross-infections in dental settings as effectively as possible [10]. As part of these infection prevention and control practices, based on published recommendations for dental patient care, patients used a 1% hydrogen peroxide mouth rinse at the beginning of the treatment [10]. However, evidence on the virucidal efficacy of mouth rinses has been fast emerging since COVID-19 became a global threat. In the meantime, the virucidal effect of hydrogen peroxide has been called into question [19, 20]. Laboratory evidence suggests that alternative mouth rinses containing polyvidone-iodine, dequalinium chloride and benzalkonium chloride, or ethanol and essential oils are superior in reducing the viral infectivity of nasopharyngeal secretions [21]. This emphasizes the importance of regularly reviewing infection prevention and control practices in the light of currently available evidence. Such scientific scrutiny is vital to ensure that the most effective infection prevention and control measures are implemented—and adapted if required—in a timely manner.

Advanced PPE, including face shields and N95 respirators (or equivalent respirators), offers a high level of protection for DHCP even during splatter and aerosol-generating procedures [3]. The wearing of masks was mandated at the UZB for staff and patients alike, and DHCP involved in the delivery of patient care were equipped with KN95 respirators (one respirator per shift) [10]. It is, however, crucial to consider that PPE procurement may be challenging during a global health crisis [22]. The targeted allocation of appropriate PPE is therefore of paramount importance for dental healthcare providers. Reuse of respirators may alleviate the problem of PPE scarcity, but further research into safe and cost-effective decontamination procedures is required [23].

This retrospective study has some limitations that need consideration. First, the dataset contained no information on SARS-CoV-2 infections among DHCP, and data on SARS-CoV-2 infections among patients were based on self-reports and a screening that did not involve virological testing. Consequently, this study precludes any conclusion about the impact of the precaution measures regarding cross-infections at the dental emergency service.

Second, the retrospective study examined data from a single major dental institution in Switzerland. Its findings should be interpreted in the context of demographic aspects, dental public health, and the government response to the COVID-19 pandemic because these factors likely affect the demands that dental emergency services face.

Third, thanks to rapid advances in COVID-19 research, the list of underlying medical conditions considered to increase the risk for severe illness from COVID-19 has seen some changes over the past months. The list of underlying medical conditions used during data extraction for this study was not exhaustive and it reflects the known risk factors at that time rather than those currently known. For example, no information on patients’ body mass index or other indicators for overweight or obesity was acquired in their standard medical history.

Fourth, no data were gathered on patients’ acceptance of the special precaution measures or on characteristics of their dental health seeking prior to their visit to the UZB. Further research focusing on the perspective of patients is required to gain insight into patients’ experience of seeking and receiving dental care during health crises such as the COVID-19 pandemic. In addition, survey or focus group data are needed to assess and draw lessons from the experience of DHCP delivering dental care under such unprecedented circumstances.

## Conclusions

Within the limitations of this retrospective study, it can be concluded that the dental emergency service faced different demands at distinct phases of the COVID-19 pandemic. The nationwide lockdown significantly impacted the dental emergency service in terms of patients’ treatment needs and the characteristics of the urgent dental care that was delivered. Persons with comorbidities changed their dental care-seeking behavior during lockdown and beyond. During health crises such as the COVID-19 pandemic, which put restrictions on dental healthcare services, it is therefore important to monitor and confirm equitable access to essential dental care.
